# Not All Is Lost for Relapsers: Relapsers With Low WHO Risk Drinking Levels and Complete Abstainers Have Comparable Regional Gray Matter Volumes

**DOI:** 10.1111/acer.14377

**Published:** 2020-06-17

**Authors:** Dieter J. Meyerhoff, Timothy C. Durazzo

**Affiliations:** ^1^ From the Center for Imaging of Neurodegenerative Diseases (CIND) (DJM) San Francisco VA Medical Center San Francisco California; ^2^ Department of Radiology and Biomedical Imaging (DJM) University of California San Francisco California; ^3^ Mental Illness Research and Education Clinical Centers (TCD) VA Palo Alto Health Care System Palo Alto California; ^4^ Department of Psychiatry and Behavioral Sciences (TCD) Stanford University School of Medicine Stanford California

**Keywords:** Alcohol Relapse, Treatment Outcome, Nonabstinent Recovery, WHO Risk Drinking Level, Brain Gray Matter

## Abstract

**Background:**

Reductions in substance use are associated with positive long‐term treatment outcomes such as psychosocial functioning; substance use–related consequences; and mental, physical, and neurobiological health. Therefore, nonabstinent clinical trial endpoints have received growing attention from substance abuse treatment experts. Regional brain tissue volumes in alcoholism treatment seekers increase during abstinence, in parallel to cognitive performance.

**Methods:**

We examined the relationships of drinking levels in those who did not maintain abstinence after treatment with magnetic resonance imaging‐derived gray matter (GM) volumes measured 8 months after baseline assessments while in outpatient treatment. The complex drinking behavior during the interval was operationalized as World Health Organization risk drinking levels (WHO‐RDL), derived from the number of standard alcoholic drinks per day. We compared the volumes of addiction‐relevant cortical and subcortical brain regions at long‐term follow‐up among abstainers and 2 groups of relapsers with low and higher WHO‐RDL.

**Results:**

We found that: (i) relapsers with low WHO‐RDL at follow‐up, who as a group had reduced their risk levels by 2.8 units (by consuming <40 g of ethanol per day over the recovery interval), had regional brain tissue volumes indistinguishable from those of abstainers tested after the same time period and that (ii) relapsers with higher WHO‐RDL, with only 1.2 units average risk‐level reductions, had significantly smaller frontal GM and thalamic volumes than abstainers and relapsers with low WHO‐RDL. Despite many drinking days during recovery, including several with >10 drinks per day, relapsers with low WHO‐RDL at follow‐up tended to perform better than those with higher WHO‐RDL on cognitive domains of working memory and visuospatial skills assessed over the recovery interval.

**Conclusions:**

Nonabstinent recovery is characterized by addiction‐relevant GM volumes comparable to those of complete abstainers. The WHO‐RDL has meaningful structural neuroimaging correlates potentially suitable as cognitively relevant biomarkers of treatment response and general brain health, perhaps even as objective clinical trial endpoints.

## Graphical abstract

Non‐abstinent recovery after treatment for alcohol use disorder (AUD) has been shown to be beneficial for psychological and physical health. Here, researchers demonstrate by MRI that non‐abstinent recovery also benefits frontal gray matter volume, a brain region important for cognitive control. Treated AUD individuals have smaller volumes than healthy controls (CON), but the volumes are similar for total abstainers (ABST) and relapsers with low drinking severity (WHO low). Only severe relapsers (WHO higher) show dramatically less frontal gray matter.

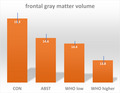

Alcohol use disorder (AUD) is associated with significant brain atrophy indicated by quantitative magnetic resonance imaging (MRI; i.e., both cortical and subcortical gray matters (GM) as well as white matter volume losses) and with multiple cognitive deficits, which both are related and may contribute to greater relapse risk after treatment (for review, see, e.g., Meyerhoff, 2020; Zahr, 2014). Particularly, tissue loss and other abnormalities in the frontal lobes have been related to greater relapse propensity. Longitudinal neuroimaging research by us and others has shown significant brain tissue volume increases with short‐ and long‐term abstinence (for review, see Meyerhoff, 2020; Zahr, 2014); measurable volume increases have been observed after as few as 2 weeks of abstinence (Durazzo et al., 2015; Gazdzinski et al., 2005; Mon et al., 2011; van Eijk et al., 2013). We showed specifically that, during treatment over approximately 5 weeks, individuals who maintained sustained abstinence over 12 months after treatment demonstrated significantly greater anterior frontal GM volume increases than those who relapsed following treatment (Durazzo and Meyerhoff, 2017, 2019; Durazzo et al., 2015); in other analyses, we found that the volumes of striatal, thalamic, GM, and white matter (WM) structures in AUD treatment seekers recovered significantly over 8 months of abstinence, albeit at different rates (Durazzo et al., 2015; Zou et al., 2018). In addition to brain tissue volume increases, abstinence was associated with better physical and mental health, improved quality of life, and lower future relapse risk (see, e.g., reviews on cognitive improvements and/or recovery of brain functions [Meyerhoff, 2020]); accordingly, brain tissue volume gains were associated with cognitive improvements in multiple domains (e.g., Durazzo et al., 2015; van Eijk et al., 2013).

While abstinence is currently the most common goal of most AUD and substance use disorder treatments, many treatment seekers struggle with the expectation of complete abstinence and may be better motivated by nonabstinence goals (drinking reduction) than strict abstinence (e.g., DeMartini et al., 2014; Haug et al., 2018). Consequently, various levels of substance use are commonly observed during and after substance use disorder treatment. The AUD treatment communities and clinical investigators have started to discuss to what degree reduced drinking after treatment can be considered a treatment success or associated with adaptive psychosocial functioning (e.g., Falk et al., 2019; Witkiewitz, 2013). Recent evidence suggests that in addition to abstinence, reductions in substance use are also associated with positive long‐term treatment outcomes such as psychosocial functioning, mental and physical health, and reduced substance use–related consequences (Falk et al., 2010; Hasin et al., 2017; Kline‐Simon et al., 2013; Miguel et al., 2019; Roos et al., 2019a, 2019b; Witkiewitz et al., 2018a, 2017b). For these and other reasons (see, e.g., Falk et al., 2019), nonabstinent alcohol and other substance use disorder clinical trial endpoints have received growing attention from regulatory agencies and clinical researchers. Examples of such endpoints for AUD treatment are number of heavy drinking days, percent days drinking, standard drinks per day or drinking day, and the World Health Organization defined risk drinking levels (WHO‐RDL) that are based on daily alcohol consumption (World Health Organization, 2000). The WHO criteria classify relapsers according to 4 alcohol risk levels (very high, high, moderate, and low) that are based on gender‐specific daily alcohol consumption averaged over a time period, such as during or after treatment (e.g., Witkiewitz et al., 2018b). Reduced WHO‐RDL during treatment relative to pretreatment levels have been associated with better psychosocial functioning/physical health/quality of life (Witkiewitz et al., 2018b) and lower alcohol‐related consequences (Witkiewitz et al., 2017a, 2019a). In Europe, a 2‐level reduction in WHO‐RDL is considered an official clinical trial endpoint criterion (European Medicines Agency, 2000).

Reflecting the focus of most current treatment programs on complete and sustained abstinence, it is not surprising that neuroimaging studies for the objective assessment of clinically relevant brain neurobiology during recovery from substance use have focused on those who maintain complete abstinence. As it is expected that sustained abstinence is related to the greatest neurobiological change in neuroimaging studies, relapsers have been commonly excluded from clinical studies that aim to measure neurobiological changes in treatment. Consequently, neuroimaging data on nonabstinent recovery are extremely sparse and the degree to which reduced alcohol consumption after treatment—as opposed to complete abstinence—is related to neurobiological recovery is unclear. In this context, there is some evidence that low‐level alcohol consumption after AUD treatment (i.e., nonabstinence) is associated with brain structural improvements: A few relapsers who had consumed up to 10 g of pure ethanol (EtOH) per day averaged over 6 months demonstrated measurable volume increases in cortical, striatal, and WM regions (Segobin et al., 2014). Within the cocaine use disorder literature, a quantitative neuroimaging study described GM volume recovery in abstinent treatment seekers with cocaine use disorder and also reported that several lapses over the 6‐month follow‐up period did not necessarily derail prefrontal cortex volume recovery (Parvaz et al., 2017). Similarly, neuroimaging outside the treatment setting in the context of harm reduction has rarely been performed in individuals with substance use disorder interested in reducing their substance use. However, a recent study showed that voluntarily reducing, rather than ceasing, cocaine consumption over several months and outside a formalized substance use disorder treatment setting was related to significant structural improvements in prefrontal GM, which correlated with improvements in executive functioning (Hirsiger et al., 2019).

In this secondary data analysis project, we used quantitative MRI data from relapsed AUD treatment seekers that had been previously acquired and processed as part of our research describing brain volume recovery in abstainers (Durazzo et al., 2017; Zou et al., 2018). Here, we examined relationships between drinking levels in those who did not maintain abstinence after AUD outpatient treatment and MRI‐derived GM volumes measured approximately 8 months after baseline assessments early in treatment. Through repeated timeline follow‐back (TLFB) interviews, we assessed the pattern of alcohol consumption following treatment and sought to distill a meaningful parameter that might capture the complex drinking behavior during the interval. We postulated that WHO‐RDL would constitute such a meaningful measure, and we tested 2 main hypotheses specifically related to frontal cortical and subcortical GM volumes measured around 8 months after baseline assessments:

1. Relapsers with low WHO‐RDL over the period following outpatient treatment (REL^low^) have GM volumes at the end of the 8‐month period that is equivalent to those of abstainers (ABST) after the same time period.
2. The corresponding GM volumes of relapsers with higher‐risk levels (REL^higher^) are significantly smaller than those of ABST.

## Materials and Methods

### Participants and Assessments

The presented research data were obtained as part of a study that was approved by the Committee on Human Research of the University of California San Francisco and the San Francisco Veterans Affairs Medical Center (SFVAMC). Prior to study participation, all participants provided written informed consent per the Declaration of Helsinki. Treatment seekers with AUD were recruited from the SFVAMC Substance Abuse Day Hospital and the San Francisco Kaiser Permanente Chemical Dependence Recovery outpatient treatment clinics and enrolled for a study of neurobiological and neurocognitive changes during and after AUD treatment.

At study enrollment, all participants were between 18 and 60 years old. At approximately 1 week into AUD treatment (i.e., after severe withdrawal symptoms had subsided), they completed the Structured Clinical Interview for DSM‐IV Axis I Disorders (Patient Edition, Version 2.0) to rule out candidates with potential neuropathology that can affect brain morphology, a neurocognitive battery assessing multiple cognitive domains of functioning known to be adversely affected by AUD (executive function, cognitive efficiency, processing speed, working memory, auditory verbal learning and memory, visuospatial skills, and fine motor skills; Durazzo et al., 2007), the Lifetime Drinking History (LDH) interview (Sobell and Sobell, 1990; Sobell et al., 1988), and an in‐house interview questionnaire for other substance consumption (type, quantity, and frequency) based on the Addiction Severity Index and NIDA Addictive Drug Survey (Pennington et al., 2015). Primary inclusion criteria were a DSM‐IV diagnosis of alcohol abuse or dependence, and consumption of more than 150 (80 for females) standard alcoholic drinks (containing approximately 14 g of pure EtOH) per month for at least 8 (6 for females) years prior to enrollment for male participants and with no dependence on substances other than alcohol or nicotine within 5 years prior to enrollment. Other medical and psychiatric exclusion criteria for the treatment seekers were detailed previously (Durazzo et al., 2015). Briefly, those with any medical and/or psychiatric condition known to influence study outcome measures were excluded except those with hepatitis C, type 2 diabetes, hypertension, unipolar mood disorders, and cigarette smoking as these conditions are highly comorbid in AUD (see Durazzo et al., 2015, and references therein). All participants also completed standardized questionnaires that assessed depression (Beck Depression Inventory, BDI) and state‐trait anxiety (State‐Trait Anxiety Inventory, STAI) symptoms.

The LDH interview at baseline yielded average number of standard alcohol‐containing drinks consumed per month over lifetime and over 1 year prior to enrollment. The individual LDH data were also used to calculate the individual’s pretreatment WHO‐RDL. At follow‐up, which was on average approximately 8 months after baseline, an in‐person TLFB Interview (Sobell and Sobell, 1992; Sobell et al., 1988) was conducted generally within 3 days of the MRI scan to assess for any alcohol consumption or other substance use since treatment initiation. Those who self‐reported complete abstinence over the 8‐month interval and had no medical record report of any alcohol consumption were designated abstainers (ABST); those who self‐reported any alcohol use or had medical records clearly indicating any alcohol consumption or relapse after baseline procedures were classified as relapsers (REL). For the purpose of this analysis, we included 28 REL and 26 ABST matched on age (all ABST older than the oldest REL participant (60 years) were excluded), sex, and current smoking status. The REL group was further divided into 2 groups based on their WHO‐RDL calculated from their TLFB alcohol consumption data averaged over the entire recovery period, akin to previous approaches (e.g., Hasin et al., 2017). Also, at follow‐up approximately 8 months after baseline, we repeated the cognitive testing as well as BDI and STAI questionnaires.

While we described the effects of 8 months of abstinence on longitudinal brain structure and cognition in previous reports (Durazzo and Meyerhoff, 2020; Durazzo et al., 2015, 2007), here, we describe cross‐sectionally brain structure and neurocognition in individuals who had relapsed at some point between their initial assessment in outpatient AUD treatment and their MR study approximately 8 months later. To test our a priori hypotheses, we compared brain structural measures from the relapsers at 8 months to those of complete abstainers at 8 months and related the imaging measures to WHO‐RDL and other relapse drinking metrics as well as neurocognitive scores obtained contemporaneously.

### MRI Acquisition and Processing

As described in our previous report on structural recovery in ABST (Durazzo et al., 2015), all study participants, abstinent or relapsed, had an MR examination approximately 8 months after a baseline examination at the beginning of AUD treatment (this baseline MRI is not used in the current analyses). T1‐weighted structural MR images were acquired orthogonal to the long axis of the hippocampus using a 3‐dimensional Magnetization‐Prepared Rapid Gradient‐Echo (MRPAGE) sequence with 1 × 1 × 1.5 mm^3^ resolution on a 1.5 Tesla Siemens scanner (Siemens Medical Systems, Iselin, NJ); the TR/TE/TI was 10/4/300 ms. Tissue intensity–based segmentation of cortical and subcortical GM, WM, and cerebrospinal fluid (CSF) used the semi‐automated probabilistic expectation–maximization segmentation method (Mon et al., 2014; Van Leemput et al., 1999). Absolute volumes (in cc) for bilateral GM and WM of the 4 major lobes and bilateral subcortical regions (cerebellum, thalamus, caudate, and lenticular nucleus [sum of putamen and globus pallidus]) and ventricles were obtained by nonlinear coregistration of tissue maps to a reference atlas (see Studholme et al., 2003, for method details and reliability). Total cortical GM and WM volumes were calculated by summing the respective GM and WM volumes from the 4 major lobes. Values for each region of interest represent the average of left and right hemispheres, as we observed no significant hemisphere differences. The regional volumes are expressed as % of intracranial volume (ICV), which was calculated as the sum of total GM, WM, and ventricular and sulcal CSF volumes, and accounts for head size differences between male and female participants.

### Statistical Analyses

We present analyses of ICV‐normalized regional brain volumes obtained 8 months after AUD treatment cessation in 28 REL, who had relapsed to varying degrees of alcohol consumption within the previous 8 months. The individuals were grouped by WHO‐RDL (based on gender‐specific drinks per day averaged over the 8 months following treatment). Cross‐sectional regional brain volumes from relapsers with low‐risk (REL^low^), pooled higher‐risk WHO levels (REL^higher^), and a group of age‐ and gender‐matched abstainers (ABST) were compared.

Analyses used independent‐samples *t* tests and chi‐square tests (or Fisher’s exact probability tests where indicated) to compare the groups on demographics, and behavioral and cognitive measures and univariate analysis of variance (ANOVA) with pairwise comparisons to compare the 3 groups on volumetric outcome measures. Based on our *a priori* hypotheses, the primary outcomes tested were frontal GM and 4 subcortical GM volumes (thalami, caudate, lenticular nuclei, and cerebellum). As the recovery interval differed by group (see below) and brain tissue volumes generally recover after cessation of drinking as a function of time, we used the recovery interval as a covariate in general linear modeling, after verifying linearity between the covariate and dependent variables, and after having demonstrated the absence of a significant interaction between covariate and group. Spearman’s correlations were used to examine associations of regional brain outcome measures with cognitive domain scores and WHO‐RDL. A significance level of *p* < 0.05 was considered statistically significant for main effects, group pairwise comparisons, and correlational analyses.

## Results

### Participants and WHO Risk Drinking Levels (WHO‐RDL)

Included in this cross‐sectional analysis were 28 REL and 26 ABST; the groups were well‐matched on age, education, sex, the proportion and severity of cigarette smoking, length of recovery interval, and lifetime drinking histories. The 2 groups also did not differ significantly on basic mood measures (see Table 1).

**Table 1:** Demographics and LDH by Relapse Status Group

Measure	REL	ABST
*N* (m/f)	28 (25/3)	26 (24/2)
Smoker (%)	54	54
Age at study enrollment (years)	47.3 ± 7.4	47.0 ± 9.8
Education (years)	13.9 ± 2.1	13.7 ± 1.8
Recovery interval (days)	226 ± 64	223 ± 48
Measures related to lifetime alcohol use history		
1‐year average (drinks/month)	454 ± 235	427 ± 254
Lifetime average (drinks/month)	250 ± 175	222 ± 121
Lifetime years	29.8 ± 8.7	30.6 ± 10.4
Onset of heavy drinking (years)	24.5 ± 8.7	23.9 ± 8.8
Duration of heavy drinking (years)	19.5 ± 9.3	19.6 ± 8.6
1‐year drinks per day	15.1 ± 7.8	14.2 ± 8.5
Measures related to smoking and mood at follow‐up		
FTND total score	4.7 ± 2.7	5.1 ± 1.7
Cigarettes smoked per day	18.4 ± 13.5	18.0 ± 5.9
BDI	9.9 ± 8.7	6.6 ± 4.3
State‐Trait Anxiety Index_Y1	36.5 ± 12.2	32.9 ± 7.7
State‐Trait Anxiety Index_Y2	43.8 ± 11.5	39.8 ± 10.7

Relapsers (REL) and abstainers (ABST) did not differ significantly on any of these measures.

BDI, Beck Depression Inventory; LDH, Lifetime Drinking History; FTND, Fagerstrom Test for Nicotine Dependence.

The REL group was further subdivided according to the WHO‐RDL: 17 relapsers with low WHO‐RDL at 8 months (REL^low^) had consumed on average <40 g of alcohol per day over 8 months (<20 g for women); they had reduced their risk from pretreatment levels by 2.8 ± 0.5 WHO‐RDL on average (15 decreased by 3 levels, 1 each by 1 and 2 levels). Eleven relapsers with higher WHO‐RDL at 8 months (moderate, high, and very high; REL^higher^) had drunk on average >40 g of alcohol per day over 8 months (>20 g for women); as a group, they had reduced their risk from pretreatment levels by only 1.2 ± 0.9 WHO‐RDL (5 reduced their level by 2, 3 by 1 level, and 3 did not change their level; *p* < 0.00001).

Table 2 lists basic demographics, LDH data, and TLFB alcohol consumption details for these 2 REL subgroups. Compared to REL^low^, REL^higher^ had a higher number of monthly alcoholic drinks averaged over lifetime and were studied after a longer recovery interval (both *p* < 0.05); REL^higher^ also tended to drink for longer (lifetime years) and drink for longer at levels above 100 dri/month (both *p* < 0.1) than REL^low^ before AUD treatment. Consistent with their different WHO‐RDL, the groups differed significantly on several alcohol consumption measures during the recovery interval (see Table 2). However, the groups did not differ significantly on their number of drinks per drinking day, their time to first relapse drink (or slip; aka time of initial sobriety after formalized treatment), and their abstinence duration before MRI (all *p* > 0.21). These latter points are important, as it suggests that the groups’ regional brain volumes at follow‐up are likely not affected differentially by the time since last alcohol‐containing drink (i.e., duration of abstinence before MRI) or the number of drinks on an average drinking day, but rather by the cumulative alcohol consumption (number or % of drinking days and amount of alcohol consumed overall or per day, as expressed in the WHO‐RDL) and by the overall length of abstinence or duration of relapse during the entire recovery period. Appendix S1 has more information about the exploratory analyses with these potential determinants of regional GM brain volume that led us to this assertion, in both text and table formats (see Table S1).

**Table 2:** Demographics, LDH, Relapse Drinking, and Cognitive Performance by WHO‐RDL Group (*p*‐Values Are From Fisher’s Exact, Chi‐square, or Student’s *t* tests)

Measure	REL^low^	REL^higher^	*p*
*N* (m/f)	17 (16/1)	11 (9/2)	0.543
Smoker (%)	53	55	0.923
*n* with medical comorbidities (hypertension/hepatitis C)	3 (2/2)	5 (3/3)	0.421
Age (years)	45.9 ± 8.4	49.3 ± 5.2	0.241
Education (years)	14.1 ± 2.3	13.7 ± 1.8	0.582
Recovery interval (days)	205 ± 70	258 ± 38	**0.031**
Measures related to lifetime drinking history			
1‐year average (drinks/month)	411 ± 228	519 ± 242	0.244
Lifetime average (drinks/month)	197 ± 96	331 ± 236	**0.045**
Lifetime years	27.6 ± 8.9	33.2 ± 7.4	0.094
Onset of heavy drinking (years)	26.5 ± 10.1	21.2 ± 4.9	0.118
Duration of heavy drinking (years)	17.0 ± 9.3	23.3 ± 8.4	0.078
1‐year drinks per day	13.7 ± 7.6	17.3 ± 8.1	0.244
Measures related to lifetime smoking history			
FTND total score	5.1 ± 2.9	4.2 ± 2.3	0.521
Cigarettes smoked per day	18.7 ± 13.6	18.0 ± 14.6	0.929
Measures related to recovery interval			
Change in WHO‐RDL over interval (range)	2.8 ± 0.5 (−1 to −3)	1.2 ± 0.9 (0 to −2)	**0.000**
Abstinence duration before MRI (days)	33 ± 48	56 ± 67	0.308
Duration of abstinence after baseline			
Assessment (days)	122 ± 104	129 ± 135	0.881
Alcohol use duration (days)	35 ± 53	113 ± 65	**0.002**
% days drinking (range)	19 ± 26 (1 to 83)	39 ± 18 (18 to 82)	**0.037**
Number of drinks per month (range)	20 ± 18 (1 to 68)	155 ± 77 (78 to 325)	**0.000**
Number of drinks per day (range)	1.0 ± 1.0 (0.1 to 2.3)	5.2 ± 2.6 (2.6 to 10.8)	**0.000**
Number of drinks per drinking day (range)	11 ± 9 (1 to 33)	15 ± 7 (6 to 24)	0.216
Number of total drinks (range)	137 ± 148 (4 to 594)	1475 ± 750 (629 to 3,024)	**0.000**
Percent of pretreatment drinking^b^ (range)	7 ± 6 (0.1 to 23)	37 ± 17 (15 to 69)	**0.000**
Select cognitive domain *z*‐scores			
Working memory at baseline	0.12 ± 0.84	−0.57 ± 0.73	**0.042**
Working memory at follow‐up	−0.03 ± 0.81	−0.65 ± 0.94	0.073
Visuospatial skills at baseline	−0.01 ± 0.91	−0.59 ± 0.69	0.094
Visuospatial skills at follow‐up	0.13 ± 1.03	−0.60 ± 0.74	0.063
Visuospatial learning at baseline	−0.54 ± 1.26	−0.82 ± 1.14	0.552
Visuospatial learning at follow‐up	−0.58 ± 1.13	−1.38 ± 1.07	0.072

LDH, Lifetime Drinking History.

Statistically significant p‐values are bolded.

^a^ Based on drinks/month over assessment interval divided by drinks/month over 1 year before treatment.

### Brain Volume Data

A 3‐group ANOVA (REL^low^, REL^higher^, and ABST) showed that the recovery interval showed a trend for group (*p* = 0.052), and *post hoc* tests showed that REL^higher^ had a longer recovery period in this study than REL^low^ (*p* = 0.016) and ABST (*p* = 0.082). Since it is widely documented that brain tissue volumes generally increase with abstinence, we used the recovery interval as a covariate in all subsequent statistical analyses of regional brain volume data. ICV‐normalized regional brain volumes by group and *p*‐values for the ANCOVA and *post hoc* tests are given in Table 3: Frontal GM was significantly different between groups (*p* = 0.002); *post hoc* tests showed that REL^higher^ had smaller frontal GM volumes than REL^low^ (*p* = 0.039, Cohen’s *d* = 0.75) and ABST (*p* = 0.001, Cohen’s *d* = 1.18), with corresponding moderate‐to‐large effect sizes for these mean differences; frontal GM volumes were not statistically different between REL^low^ and ABST (*p* = 0.152, Cohen’s *d* = 0.53). The GM volumes of the parietal, temporal, and occipital lobes did not differ significantly between groups (all *p* > 0.42, Cohen’s *d* < 0.43), highlighting the well‐established adverse consequences of AUD on the frontal GM. Total GM volume (as a sum of all GM lobe volumes) differed between the groups (*p* = 0.042), and *post hoc* tests indicated smaller volumes in REL^higher^ compared with both REL^low^ (*p* = 0.044, Cohen’s *d* = 0.74) and ABST (*p* = 0.014, Cohen’s *d* = 0.77); however, these group differences in total GM volume were largely driven by the differences in frontal GM. In addition, the volume of the bilateral thalami was significantly different between the groups (*p* = 0.042); *post hoc* tests showed thalamic volume was significantly smaller in REL^higher ^compared with REL^low^ (*p* = 0.013, Cohen’s *d* = 0.75) and tended to be smaller relative to ABST (*p* = 0.051, Cohen’s *d* = 0.56). Caudate and lenticular GM volumes did not differ significantly between groups (both *p* > 0.27, Cohen’s *d* < 0.46). The thalamic volume group difference did not survive multiple comparison correction for the 4 subcortical regions tested.

**Table 3:** Regional gray matter (GM) volumes by group as % of intracranial volume (*F*‐statistics and follow‐up *post hoc* tests from 3‐group ANCOVA with recovery interval as covariate; pairwise comparisons are Bonferroni‐corrected)

Measure	ABST *n* = 26	REL^low^ *n* = 17	REL^higher^ *n* = 11	ANCOVA	*p* REL^low^ versus REL^higher^	*p* REL^low^ versus ABST	*p* REL^higher^ versus ABST
Frontal GM	14.8 ± 0.8	14.4 ± 0.7	13.8 ± 0.9	***F*(2, 51) = 6.97, *p* = 0.002**	**0.039**	0.152	**0.001**
Temporal GM	9.9 ± 0.5	9.7 ± 1.2	9.5 ± 0.7	0.422			
Parietal GM	8.0 ± 0.5	8.4 ± 1.4	8.0 ± 0.7	0.480			
Occipital GM	3.6 ± 0.4	3.6 ± 0.4	3.5 ± 0.5	0.739			
Total GM	36.4 ± 1.5	36.2 ± 1.1	34.9 ± 2.4	***F*(2, 51) = 3.39, *p *= 0.042**	**0.044**	0.823	**0.014**
Thalamus GM	0.41 ± 0.09	0.42 ± 0.07	0.36 ± 0.09	***F*(2, 52) = 3.38, *p* = 0.042**	**0.013**	0.306	**0.051**
Caudate GM	0.45 ± 0.06	0.45 ± 0.05	0.46 ± 0.05	0.788			
Lenticular GM	0.42 ± 0.06	0.44 ± 0.09	0.44 ± 0.07	0.277			

Statistically significant p‐values are bolded.

### Correlations of GM Volumes With WHO‐RDL and Other Measures of Drinking During Recovery Interval

**Fig. 1:**
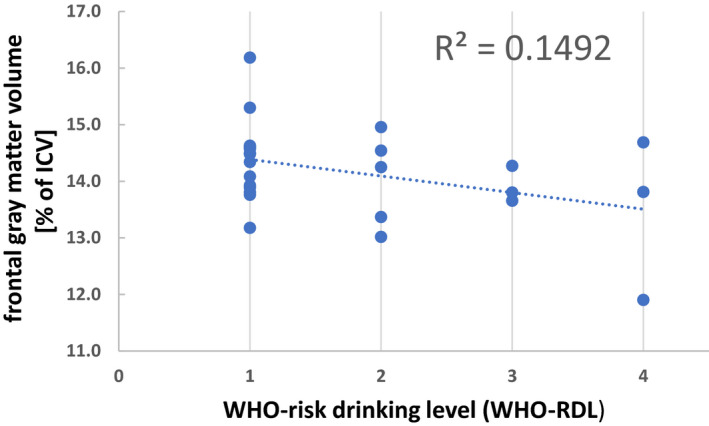
Correlation of normalized frontal gray matter volume (as % of intracranial volume, ICV) with WHO risk drinking level (WHO‐RDL) in relapsed treatment seekers (REL^low^ and REL^higher^ combined) at follow‐up. Spearman’s *ρ* = −0.37, *p* = 0.056. WHO‐RDL 1 = low, 2 = moderate, 3 = high, 4 = very high.

WHO‐RDL in REL (REL^low^ and REL^higher^ combined) at follow‐up tended to correlate inversely and moderately strong with frontal GM at follow‐up (Spearman’s *ρ* = −0.37, *p* = 0.056; see Fig. 1) and with overall GM volumes (Spearman’s *ρ* = −0.36, *p* = 0.061), consistent with the group comparisons described above. Although not statistically significant, these correlations with WHO risk levels were stronger than with the constituting measure “drinks per day during interval” (both Spearman’s *ρ* < −0.22). Total GM (Spearman’s *ρ* = −0.35, *p* = 0.070) and frontal GM (Spearman’s *ρ* = −0.33, *p* = 0.104) in REL also tended to correlate inversely with “percent of 1‐year monthly (pretreatment) drinking.”

### Further Drinking and Cognition Characteristics of REL Subgroups

Estimated over the 8 months after treatment cessation, REL^low^ had an average monthly alcohol consumption of 7 ± 6% of their corresponding 1 year pretreatment levels (vs. 37 ± 17% in REL^higher^), and they had an alcohol use duration of 35 ± 53 days (vs. 113 ± 65 days in REL^higher^) with an average of 20 ± 18 standard alcoholic drinks per month (vs. 155 ± 77 drinks in REL^higher^; all *p* < 0.005; see Table 2). REL^low^ did not differ from REL^higher^ on the number of drinks per drinking day, which was quite substantial for both groups (11 ± 9 vs. 15 ± 7, respectively, *p* = 0.216), with some REL^low^ participants consuming between 20 and 33 drinks on the days they drank. All of the individuals in REL^higher^ had an average daily alcohol consumption that matched the definition of heavy drinking days (>4 drinks per day in women, >5 drinks per day in men), whereas 12 of the 17 REL^low^ participants consumed on average in access of the heavy drinking day lower limits. The exact number of heavy drinking days in the interval could not be calculated from our TLFB interview data, as we did not perform the fine‐grained evaluation commonly done for shorter survey periods often associated with treatment studies. However, the other TLFB data obtained suggested that despite several heavy drinking days in the recovery interval, the frontal GM volumes of individuals after 8 months of nonabstinent recovery were statistically equivalent to those observed in complete abstainers.

Regarding cognitive performance, REL^low^ tended to perform better than REL^higher^ on domain scores for working memory and visuospatial skills across the assessment interval (at baseline: *p* < 0.045 and <0.095, respectively; at follow‐up: both *p* < 0.075), and both domain scores correlated positively with the frontal GM volumes of the combined group of relapsers at follow‐up (Spearman’s *ρ* = 0.42, *p* = 0.036 for visuospatial skills; Spearman’s *ρ* = 0.37, *p* = 0.066 for working memory). The 2 WHO subgroups did not differ significantly at either baseline or follow‐up on the domain scores for executive function, cognitive efficiency, processing speed, auditory verbal learning and memory, fine motor skills (all *p* > 0.20), and intelligence (*p* > 0.11).

## Discussion

The recent interest in harm reduction from reduced substance use and in clinical trial endpoints other than complete abstinence raises the question how the brain morphology of treatment seekers who do *not achieve complete abstinence* compares to that of complete abstainers or full relapsers. Using previously acquired and processed neuroimaging data in treatment‐seeking individuals with AUD, we analyzed regional GM volumes, which are putative/objective measures of functionally/cognitively relevant brain health, as a function of WHO risk drinking levels (WHO‐RDL). The WHO‐RDL was calculated based on gender‐specific daily alcohol consumption averaged over approximately 8 months following a baseline assessment early in AUD treatment. During this relatively long period, our study participants experienced different temporal relapse patterns with varied quantity/frequency of alcohol consumption before they underwent a quantitative MRI research scan. We tested whether the WHO‐RDL is a meaningful determinant of cross‐sectional cortical and subcortical brain volumes measured after that rather long time period of reduced alcohol consumption. Our results indicate that, at approximately 8 months after entering AUD treatment, relapsers with low WHO‐RDL have frontal cortical brain volumes that are statistically equivalent to those of complete abstainers after the same time period and significantly larger than those of relapsers with higher WHO‐RDL. Those REL^low ^drank on average <40 g (i.e., less than approximately 3 standard drink equivalents) per day over the recovery period, they drank at pretreatment levels only on a few days, and the frequency of their heavy drinking days was relatively low. Therefore, they also spent considerably more time in remission than the REL^higher^, giving the brain a chance to recover and engage intrinsic neuroplastic processes to regain functionally relevant frontal cortical and subcortical tissues despite self‐reported and medical chart‐confirmed nonabstinence. Corresponding regional brain tissue volume increases have been observed in treatment seekers over several weeks and months of complete abstinence (for reviews, see Meyerhoff, 2020; Zahr, 2014). Alternatively, and absent real volume change measures in this cohort, we may also interpret our findings of larger brain tissue volumes to reflect an intrinsic characteristic of those who are able to reduce their drinking, with this group experiencing some kind of protective or preserving effect on brain tissue. Consistent with this interpretation of a population effect (rather than the result of volume increases), the REL^low^ also tended to have better working memory and visuospatial skills at both baseline and follow‐up than the REL^higher^.

A large body of research has demonstrated that greater cortical integrity, particularly in the anterior frontal lobe, is related to better function in multiple neurocognitive domains, less substance use, and lower risk of relapse after treatment (for review, see, e.g., Meyerhoff, 2020). Therefore, the novel findings presented here appear consistent with recent work that describes that those individuals who engage in some heavy drinking following AUD treatment (such as our REL^low^ participants, most of whom reduced their WHO‐RDL levels by 3 units) may function better than those demonstrating more severe relapse and function similarly to those who are mostly abstinent with respect to psychosocial functioning, employment, life satisfaction, and mental health (Witkiewitz et al., 2019b).

Beyond cortical regions, the thalamus was one of the subcortical structures found significantly smaller in REL^higher^ than REL^low^ at 8 months after treatment initiation, with no difference between REL^low^ and ABST at 8 months. Consistent with this thalamic finding, Segobin and colleagues (2014) observed in a preliminary study that AUD individuals with smaller thalami at treatment entry resumed heavy alcohol consumption during a 6‐month follow‐up period. Smaller thalamic volumes had previously been described in AUD (Le Berre et al., 2014; Pitel et al., 2012), in addition to thalamic volume increases over 8 months of complete abstinence (Durazzo et al., 2015). Taken together, these structural neuroimaging data suggest a critical role for the integrity of frontal GM and thalamus volumes in the ability to regulate alcohol consumption after treatment.

In an exploratory way, we evaluated other measures of drinking severity during the recovery period as to their relationship with GM volumes at the 8‐month follow‐up (see Appendix S1). These were measures with potential to affect regional GM measures as described in the alcohol neuroimaging literature. No other single measure was more strongly related to GM volume at 8‐month follow‐up than the WHO‐RDL classification scheme, not even its constituting measure “drinks per day during interval.”

### Limitations

The sample size of the current study can be considered modest (although not necessarily so for studies that require cohort maintenance), partly related to our initial focus on studying complete abstainers only and to our later inability to retain in this longitudinal research a sufficient number of individuals who had relapsed and were thought to be reliable reporters of their relapse. Future studies may be directed specifically at studying brain effects of relapse patterns prospectively, employing a segmentation method with higher spatial resolution to evaluate prefrontal brain regions (such as FreeSurfer methodology) and employing greater granularity in WHO‐RDL reporting (e.g., monthly as in Falk et al., 2019), perhaps supported by established biochemical indicators of alcohol consumption. Future research may also measure longitudinal changes in brain tissue volumes related to drinking reductions rather than providing cross‐sectional regional volume measures after varied reductions in alcohol consumption as presented here. As the research cohort was primarily recruited from within the VA healthcare system, only few women were enrolled, which precluded our ability to evaluate potential sex effects. Furthermore, the drinking measures from the LDH and the TLFB were self‐reported, thereby subject to potential recall and social desirability bias, with only the measures from the TLFB corroborated by medical chart reviews of the enrolled VA patients. These study limitations, however, are offset by several strengths: This is one of the first studies that relate drinking reduction to an objective measure of brain health as well as neurocognition, complementing previous research reports of beneficial effects of drinking reductions on potentially related mental and physical health as well as psychosocial functioning. The findings of an association between low WHO‐RDL and higher GM volume statistically equivalent to those in abstainers and related to better cognitive function also inform on the valuable concept of harm reduction and new clinical trial endpoints. Understanding the potential benefits of drinking reductions, in addition to and in contrast to complete abstinence, provides new information to the public, treatment providers, patients, clinical trial conductors, and public health officials on the benefits to brain health and related neuropsychological functioning.

## Conclusions

Other recent research has demonstrated that reductions in heavy drinking as operationalized by WHO‐RDL are both clinically relevant and a preferred treatment goal of many treatment seekers. Here, we evaluated whether reducing drinking levels as opposed to being completely abstinent is associated with regional brain morphometry as determined by quantitative structural brain imaging 8 months after outpatient treatment. Consistent with the harm reduction theory, our results suggest that significant decreases in post‐treatment alcohol consumption (i.e., nonabstinent recovery)—as demonstrated by relapsers with low WHO‐RDL over the observation period who reduced their WHO‐RDL by an average of 2.8 levels—are associated with similar regional GM volumes than observed in those who maintained complete abstinence during their 8‐month recovery period. Even relapsers with high alcohol consumption on several drinking days during the 8 months following initial treatment have frontal GM volumes comparable to those of complete abstainers. We conclude that the concept of WHO‐RDL has meaningful structural neuroimaging correlates; being also associated with cognition and substance use behavior including relapse, these objective cross‐sectional correlates may be suitable as brain biomarkers of treatment response and general brain health, perhaps even as objective clinical trial endpoints.

## Supporting information


**Appendix S1.** Exploratory analyses: Effects of different grouping variables on frontal gray matter (GM) volume differences.
