# Supplementary material for: Not All Is Lost for Relapsers: Relapsers With Low WHO Risk Drinking Levels and Complete Abstainers Have Comparable Regional Gray Matter Volumes
Source: Alcohol Clin Exp Res. 2020 Jun 17;44(7):1479–87. doi: 10.1111/acer.14377 (PMC7383772; doi:10.1111/acer.14377)
Supplement: Supplementary file 1 — Appendix S1. Exploratory analyses: Effects of different grouping variables on frontal gray matter (GM) volume differences. [file ACER-44-1479-s001.docx]

**Supplementary Material:**

*Exploratory analyses: Effects of different grouping variables on frontal gray matter (GM) volume differences*

We performed exploratory analyses to follow-up on the assertion that the relapsing groups’ brain volumes at follow-up are affected more so by the cumulative alcohol consumption during relapse and duration of relapse (i.e., the overall severity of relapse) than the duration of abstinence during the entire recovery period. In these analyses, we re-grouped the relapsers into about equal groups (n=14±1) with highly significant differences (p<0.0005) on these grouping variables according to their non-overlapping low and high values; these exploratory variables are related to pattern of alcohol consumption that also – similar to the WHO-RDL – characterize the recovery interval and could perceivably affect frontal GM volume (most measures as shown in **Table 2** for the main WHO-RDL grouping variable).

As summarized in **Table S1** below, we found that frontal GM volume as our primary test outcome only differed significantly between exploratory subgroups for the variable ‘% of days drinking during interval’ (7.0 vs. 48.5 %, higher frontal GM volume in low vs. high group at p=.043, Cohen’s *d*=.85), and it tended to differ for the variable ‘duration of relapse during interval’ (12 vs. 101 days, higher frontal GM volume in low vs. high group at p=.069, Cohen’s *d*=.75). The thalamic volume did not differ significantly between any of these subgroupings (p>.12).

The following grouping variables did not differentiate the low and high groups on the frontal GM measure (all p>.11): ‘number of drinks per average drinking day during the interval’; ‘number of total drinks during interval’; ‘percent of 1-year monthly (pretreatment) drinking’; ‘abstinence duration before MRI’; and ‘abstinence duration after baseline assessment’.

All our primary and exploratory analyses taken together suggest that the WHO-RDL grouping variable at follow-up (low vs. higher) is most strongly related to GM volume at 8-months-follow-up and therefore appears to be the most meaningful measure relating to functionally relevant GM volumes at follow-up.

**Table S1**: Effects of exploratory grouping variables on frontal GM volume differences among relapsers

| exploratory variable | low group | high group | cut-off value * | frontal GM volume difference p** |
| --- | --- | --- | --- | --- |
| % of days drinking during interval | 7.0 ± 7.2 | 48.5 ± 19.2 | 20 % | .043 |
| duration of relapse during interval [days] | 12 ± 12 | 101 ± 59 | 34 days | .069 |
| number of drinks per average drinking day during the interval | 4.5 ± 2.6 | 20.7 ± 7.9 | 10 | .114 |
| number of total drinks during interval | 92 ± 68 | 1321 ± 783 | 132 | .201 |
| % of 1-year monthly (pretreatment) drinking | 22 ± 16 | 105 ± 24 | 50 % | .895 |
| abstinence duration before MRI [days] | 77 ± 62 | 6 ± 5 | 14 days | .806 |
| abstinence duration after baseline assessment [days] | 46 ± 29 | 191 ± 70 | 100 days | .223 |

* value that divides groups with low and high exploratory measures

** by Student’s t-test
